# Glucose and Lactate Levels at Admission as Predictors of In-hospital Mortality

**DOI:** 10.7759/cureus.6027

**Published:** 2019-10-29

**Authors:** David Sotello, Shengping Yang, Kenneth Nugent

**Affiliations:** 1 Internal Medicine/Pulmonary and Critical Care Medicine, Texas Tech Health Sciences Center, Lubbock, USA; 2 Biostatistics, Pennington Biomedical Research Center, Baton Rouge, USA; 3 Internal Medicine/Pulmonary and Critical Care Medicine, Texas Tech University Health Sciences Center, Lubbock, USA

**Keywords:** blood glucose, lactate, in-hospital mortality, in-hospital outcomes

## Abstract

Objective

Glucose and lactate levels in patients at the time of admission have been studied in diverse patient groups. Some studies suggest that elevated glucose levels at admission predict worse outcomes. Elevated Lactate levels have also been reported to be directly associated with increased mortality. We wanted to determine if the combination of admission glucose and lactate levels improves the predictability of inpatient mortality and length of stay (LOS).

Methods

This is a retrospective study. We included all adult patients admitted at an academic medical center from October 1, 2015 to September 30, 2016. We collected basic clinical information, including age, gender, admission glucose and lactate levels, LOS, and mortality. We separated outcomes based on glucose and lactate levels by dividing them into quartiles. We also stratified patients based on normal lactate (<2.0 mmol/L), high lactate (2.0-4.0 mmol/L), and very high lactate (>4 mmol/L) levels; and on normal glucose (60-140 mg/dl), high glucose (140-200 mg/dl), and very high glucose (>200 mg/dl) levels.

Results

A total of 5,436 adult patients were included in our study. The median age was 58 years, and 57% of the patients were male. The median LOS was 6 days, and the overall in-hospital mortality rate was 11%. When the patients were separated in quartiles based on admission glucose values, mortality was higher in the 4th quartile (≥173 mg/dL): 14.87%, probability value (p): <0.001. When the patients were separated in quartiles based on lactate levels, the mortality was higher in the 4th quartile (≥2.23 mmol/L): 21.95%, p: 0.001. When the patients were paired according to normal, high, or very high lactate and glucose levels, the groups that had higher mortality were as follows: normal glucose/very high lactate: 32.43%; high glucose/very high lactate: 34.04%; and very high glucose and very high lactate: 39.15%. The groups with very high glucose and very high lactate had increased odds of mortality when compared with the other groups (p: <0.001).

Conclusions

Admission glucose and lactate levels provide useful information in the estimation of inpatient mortality. The LOS was shortened in the groups with higher glucose, lactate, or both. The combination of glucose and lactate levels predicted mortality better than either value alone.

## Introduction

The identification of potential clinical indicators that predict patient outcomes is very important in current medical practice [1,2]. There are multiple laboratory tests, clinical scores, and clinical indices that have different ranges of accuracy, and they may not be applicable in all clinical scenarios [1,2,3]. It has been established that elevated lactate levels and glucose levels, independently, are directly related to increased patient morbidity, length of stay (LOS), mortality, etc. [4]. These two basic laboratory tests are readily available in most institutions in developed countries. Their combined use has not been extensively studied, and we hypothesized that their combined use may enhance our ability to identify sicker patients who would require more intensive management to improve their outcomes. 

## Materials and methods

This is a single-center, retrospective study. It included all adults (≥18 years old) admitted to University Medical Center in Lubbock, Texas, from October 1, 2015 to September 30, 2016, whose admission glucose and lactate levels were recorded. Requests for laboratory tests were based on individual patient management decisions. Patients who were <18 years of age and >89 years of age were excluded. General demographic information was collected. This included age, gender, LOS, and mortality. University Medical Center is a 500-bed referral center in West Texas. 

The patients were stratified according to their lactate levels into three categories: lactate levels of <2 mmol/L; lactate levels of 2.0-4.0 mmol/L; and lactate levels >4.0 mmol/L. The respective LOS and mortality were calculated for each category. The patients were then classified according to their respective quartiles for their glucose and lactate levels, and their respective LOS and mortality were calculated. Patients were also classified according to their glucose/lactate molar ratios into quartiles, with their LOS and mortality for each quartile subsequently calculated.

Finally, the patients were arbitrarily classified according to their paired glucose and lactate levels. We determined the following categories: normal lactate: <2 mmol/L; high lactate: 2.0-4.0 mmol/L; and very high lactate: >4 mmol/L. These were paired with glucose levels classified as follows: normal glucose: 60-140 mg/dL; high glucose: 140-200 mg/dL; and very high glucose: >200 mg/dL. The respective LOS and mortality were calculated for each group.

Descriptive statistics were used to describe the characteristics of the study cohort. Categorical variables were summarized as frequencies, and continuous variables were summarized using medians and quartiles or percentiles, as appropriate. Univariate logistic regression was used to test if there is a significant association between mortality and assigned categories. Odds ratios (ORs) were calculated to evaluate these associations. Comparisons of LOS across categories were performed using the Kruskal-Wallis rank sum test. The statistically significant level was set at 0.05. Multiple testing adjustment was not performed. Analyses were performed using SAS software, Windows version 9.3 (SAS Institute, Cary, NC). 

## Results

A total of 5,436 patients were included in this study. The median age was 58 years (25th-75th percentile: 44-69 years); 57% of the patients were male. The median LOS was 6 days (25th-75th percentile: 3-11 days). The overall in-hospital mortality was 11.04%. The distribution of glucose, lactate, and glucose/lactate ratios for representative diagnoses are shown in Table 1.

**Table 1:** Distribution of glucose, lactate levels, and glucose/lactate molar ratios of most representative diagnoses *Numbers in parentheses represent the 25th and 75th percentiles

Category	Glucose median*	Lactate median*	Glucose/lactate median*
Sepsis (n = 799)	132 (105.0, 202.5)	1.8 (1.2, 2.8)	4.54 (2.75, 7.01)
Pneumonia (n = 118)	125 (103.0, 163.5)	1.3 (0.9, 1.6)	5.60 (4.05, 7.96)
Chronic obstructive pulmonary disease exacerbation (n = 159)	123 (108.0, 149.5)	1.3 (1.0, 2.1)	5.26 (3.48, 7.59)
Acute kidney failure (n = 81)	116 (92.0, 165.0)	1.4 (1.1, 1.9)	5.01 (3.62, 8.15)
Urinary tract infection (n = 28)	138 (106.8, 246.5)	1.1 (0.9, 1.7)	6.74 (5.13, 10.19)
Gastrointestinal bleed (n = 55)	121 (98.5, 162.5)	1.7 (1.2, 2.6)	4.69 (2.19, 8.01)
Coronary artery disease (n = 191)	118 (95.0, 149.0)	1.3 (1.0, 1.7)	5.17 (3.62, 7.32)
Acute heart failure (n = 102)	127 (108.3, 189.5)	1.4 (1.1, 1.9)	5.39 (3.85, 7.85)
Pancreatitis (n = 30)	144 (116.5, 236.5)	1.4 (0.9, 2.1)	6.81 (4.23, 9.03)
Acute respiratory failure (n = 211)	130 (104.0, 191.5)	1.5 (1.1, 2.3)	5.11 (3.38, 7.21)

The patients were stratified according to their lactate levels. Those with a lactate level of <2.0 mmol/L had a mortality rate of 7.18%; those with a lactate level between 2.0-4.0 mmol/L had a mortality of 13.16%; and those with a lactate level of >4 mmol/L had a mortality of 36.04%. Specifically, when treated as a continuous predictor, a one-unit increase in lactate was associated with a 32% increase in the odds of mortality (p: <0.001) (Table 2).

**Table 2:** Outcomes based on lactate level ¶The distributions of patient age, LOS, and mortality differ among the lactate percentile groups *Numbers in parentheses are the 25th and 75th percentiles; **numbers in parentheses are percentage figures

Lactate (mmol/L)	Lactate level: <2.0	Lactate level: 2.0–4.0	Lactate level: >4.0	All	P-value¶
Number	3,763	1,193	480	5,436	
Male/female	2,105/1,658	707/486	284/196	3,096/2,340	0.077
Median age, years*	60 (47, 70)	55 (39, 67)	56 (42, 67)	58 (44, 69)	<0.001
Median LOS, days*	6 (4, 11)	6 (3, 11)	5 (2, 10)	6 (3, 11)	<0.001
Mortality**	270 (7.18)	157 (13.16)	173 (36.04)	600 (11.04)	<0.001

The patients were next divided into quartiles according to the glucose levels. The mortality rate was as follows: 1st quartile (glucose: <102 mg/dl): 9.44%; 2nd quartile (glucose: 102-125 mg/dl): 9.26%; 3rd quartile (glucose: 125-173 mg/dL); 10.53%; and 4th quartile (glucose: ≥173 mg/dl): 14.87%. Specifically, when comparing the patients in quartiles 1, 2 and 3, the patients in the 4th quartile had a 68%, 71%, and 49% increase in the odds of mortality, respectively (p: <0.001). The LOS was shorter for those in the 4th quartile (p: <0.001) (Table 3).

The patients were also divided into quartiles according to the lactate levels. The mortality rate was as follows: 1st quartile (lactate: <1.05 mmol/L): 5.56%; 2nd quartile (lactate: 1.05-1.45 mmol/L): 6.83%; 3rd quartile (lactate: 1.45-2.23 mmol/L): 9.72%; and 4th quartile (lactate: >2.23 mmol/L): 21.95%. Compared with patients in quartiles 1, 2 and 3, the patients in the 4th quartile had a 377%, 284%, and 161% increase in the odds of mortality, respectively (p: <0.001). The LOS was shorter for those in the 4th quartile (p: <0.001) (Table 3).

When the patients were divided into quartiles by their glucose/lactate molar ratios, the mortality was as follows: 1st quartile (<3.3): 19.72%; 2nd quartile (3.3-5.1): 9.35%; 3rd quartile (5.1-7.5): 8.98%; and 4th quartile (>7.5): 6.11%. When comparing the patients in the 1st quartile glucose/lactate molar ratio, the patients in quartiles 2, 3, and 4 had a 58%, 60%, and 74% decrease in the odds of mortality (p: <0.001) (Table 3).

**Table 3:** Outcomes based on glucose and lactate levels in quartiles Q: quartiles; LOS: length of stay *Numbers in parentheses are the 25th and 75th percentiles; **numbers in parentheses are percentage figures

Patient details						
Glucose, mg/dl	Q1 (<102)	Q2 (102-125)	Q3 (125-173)	Q4 (>173)		P-value
Number	1,324	1,360	1,387	1,365		
Male/female	704/620	785/575	866/521	741/624		<0.001
Median age, years*	58 (43, 68)	59 (45, 71)	60 (46, 71)	57 (44, 67)		<0.001
Median LOS, days*	6 (3, 11)	6 (3, 11)	6 (4, 11)	5 (3, 10)		<0.001
Mortality**	125 (9.44)	126 (9.26)	146 (10.53)	203 (14.87)		<0.001
Lactate, mmol/L						
Lactate	Q1 (<1.05)	Q2 (1.05-1.45)	Q3 (1.45-2.23)		Q4 (>2.23)	P-value
Number	1,330	1,376	1,368		1,362	
Male/female	691/639	784/592	809/559		812/550	<0.001
Median age, years*	60 (47, 70)	60 (46, 70)	59 (45, 70)		55 (40, 66)	<0.001
Median LOS, days*	6 (4, 10)	6 (4, 11)	6 (3, 11)		5 (3, 11)	<0.001
Mortality**	74 (5.56)	94 (6.83)	133 (9.72)		299 (21.95)	<0.001
Glucose/lactate in molar ratios						
Glucose/lactate molar ratio*	Q1 (<3.3)	Q2 (3.3-5.1)	Q3 (5.1-7.5)		Q4 (>7.5)	P-value
Number	1,359	1,359	1,359		1,359	
Male/female	815/544	804/555	748/611		729/630	0.001
Median age, years*	57 (40, 67)	59 (43.5, 70)	60 (48, 71)		58 (46, 68)	<0.001
Median LOS, days*	6 (3, 12)	6 (3, 10)	6 (4, 11)		6 (3, 10)	0.621
Mortality**	268 (19.72)	127 (9.35)	122 (8.98)		83 (6.11)	<0.001

When the patients were classified according to glucose and lactate levels, the lowest mortality was in the group with normal glucose/lactate levels (6.39%); the highest mortality was in the groups with normal glucose/very high lactate (32.43%), high glucose/very high lactate (34.04%), and very high glucose/very high lactate levels (39.15%). In the normal glucose category, compared with patients in normal and high lactate groups, patients in the very high lactate group had a 603% and 212% increase in the odds of mortality (p: <0.001), respectively. In the high glucose category, compared with patients in the normal and high lactate groups, patients in the very high lactate group had a 345% and 295% increase in the odds of mortality (p: 0.001), respectively. In the very high glucose category, compared with patients in normal and high lactate groups, patients in the very high lactate group had a 897% and 290% increase in the odds of mortality (p: <0.001), respectively. In addition, patients in the very high glucose/very high lactate group had the shortest LOS (median: 4; 25th-75th percentiles: 2-8) (Table 4).

**Table 4:** Inpatient mortality comparison between glucose and lactate levels NG: normal glucose (60-140 mg/dl); HG: high glucose (140-200 mg/dl); VHG: very high glucose (>200 mg/dl) NL: normal lactate (<2 mmol/L); HL: high lactate (2.0-4.0 mm/L); VHL: very high lactate (>4.0 mmol/L) LOS: length of stay; *numbers in parentheses are the 25th and 75th percentiles; **numbers in parentheses are percentage figures

Glucose/lactate categories									
Glucose/lactate groups	NG, NL	NG, HL	NG, VHL	HG, NL	HG, HL	HG, VHL	VHG, NL	VHG, HL	VHG, VHL
Number	2,457	593	185	722	277	94	561	318	189
Male/female	1,379/1078	361/232	103/82	420/302	176/101	62/32	292/269	167/151	115/74
Age, years*	59 (46, 70)	55 (37, 67)	57 (46, 69)	63 (51, 72)	55 (38, 66)	58 (43, 70)	57 (44, 66)	53 (43, 66)	54 (40, 64)
LOS, days*	6 (4, 11)	6 (3, 11)	5 (3, 12)	6 (4, 11)	6 (3, 11)	6 (2, 14)	6 (3, 11)	5 (3, 10)	4 (2, 8)
Mortality**	157 (6.4)	79 (13.3)	60 (32.4)	75 (10.4)	32 (11.5)	32 (34)	34 (6.1)	45 (14.1)	74 (39.1)

**Figure 1:**
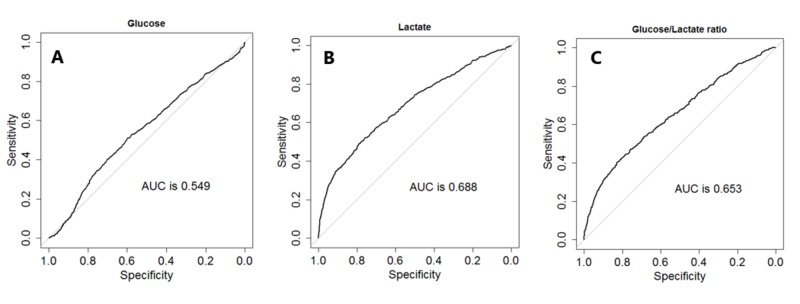
Area under the receiver operating characteristic curves (AUROCs) comparing glucose, lactate, and glucose/lactate ratio Panel A, B, and C show the AUROCs comparing the performance of glucose levels (panel A), lactate levels (panel B), and glucose/lactate ratios (panel C), showing a better performance of lactate and glucose/lactate ratios when compared with glucose alone as predictor of mortality

The area under the receiver operating characteristic curves (AUROCs) comparing glucose levels, lactate levels, and glucose/lactate ratios shows a higher AUROCs for lactate levels and glucose/lactate levels when compared to glucose levels alone, indicating better performance of lactate levels and glucose/lactate ratios for predictors for mortality (Figure 1).

## Discussion

In this study, we found that the combination of two well-known markers provides valuable information in the early identification of patients with worse outcomes. The group of patients with a combination of very high glucose and very high lactate had the highest mortality in the study cohort. Our results indicate that this combination provides better identification of the sickest patients when compared to either lactate or glucose levels alone.

It has been well established that elevated lactate levels are associated with increased morbidity, LOS, and mortality [4,5,6]. Hyperlactatemia usually occurs when lactate production exceeds lactate clearance. The most common cause is tissue hypoxia, in which lactate is the end product of glycolysis under anaerobic conditions. But this can also occur in disorders unrelated to tissue hypoxia (type B lactic acidosis) [7,8,9]. There is a direct relationship between lactate levels and poor outcomes [4]. Some studies have reported mortality between 75-100% when lactate levels exceed >10 mmol/L [7,8]. This association has been studied in several clinical scenarios, especially in septic patients, but may also be applicable in patients in other clinical settings, such as emergency room patients, patients with acute coronary syndrome, cardiogenic shock, gastrointestinal bleeding, advanced age, trauma, and postsurgical patients, etc. [3,4,10,11,12,13]. Shetty et al. found that the combination of lactate plus quick Sepsis Related Organ Failure Assessment (qSOFA) improved sensitivity in identifying adverse outcomes in suspected septic patients [1]. The optimal lactate cut-off value and the timing of lactate measurement to stratify the sickest patients are still unclear. Larger reductions from baseline lactate levels are associated with better outcomes [3,7,9,11].

The relationship between increased glucose levels and patient outcomes has also been studied, and there is evidence supporting the association of high glucose values with increased morbidity, a longer length of hospitalization, increased readmission, and worse outcomes [14,15,16,17]. Some studies suggest that these relationships exist independently of a known diagnosis of diabetes [15,18-20]. The association of hyperglycemia and worse outcomes seems to be stronger when it occurs in non-diabetic patients [16,20]. Glynn et al. found that among the patients evaluated in the emergency room, abnormal glucose values were associated with increased mortality in non-diabetic patients but not in diabetic patients [2]. Abnormally low glucose levels, especially in the ICU, have also been associated with increased mortality [2]. It is unclear if one or multiple glucose values enhance the ability to triage patients, but Haddadin et al. found that even a single admission of blood glucose could predict increased mortality at 1 year in a mixed cohort of diabetic and non-diabetic patients [19].

These associations may be related to the metabolic changes caused by an acute illness in glucose and lactate metabolism [17,21,22]. It has also been reported that multiple alterations in the glycolysis and gluconeogenesis pathways may contribute to elevated glucose levels [21-23]. Hyperglycemia per se has been associated with immunologic, metabolic, and microvascular changes [18,22]. All of these factors may contribute to a simultaneous elevation of both markers in some critically ill patients. 

Very few studies have analyzed combined glucose and lactate levels as predictors of patient outcomes. Kaukonen et al. found that there is an independent association with hyperglycemia and hyperlactatemia and increased mortality. But the association of hyperglycemia disappeared when hyperlactatemia was considered in combination, suggesting that elevated lactate levels may have a stronger association with poor outcomes [21]. Green et al. in their assessment of the association of hyperglycemia and hyperlactatemia found that mortality risk did not increase unless hyperglycemia was associated with hyperlactatemia [23]. Van Vught et al. reported that the association of hyperglycemia and mortality was lost after adjustment for lactate only in non-diabetic patients [18,22]. In our study, we did identify sicker patients when both laboratory test results were used in combination. This may be explained by the different patient populations used in these studies. The previous studies utilized these markers in emergency rooms and ICUs, respectively; we used them in all patients admitted to our hospital.

We found a decreased LOS in the groups with the highest glucose, lactate, or both. The explanation for these results is uncertain. This is in contrast to previously published studies, where elevated lactate levels have been associated with increased LOS [4]. One explanation might be the severity of illness and imminent demise; another explanation might be that these groups included a significant number of patients with diabetic ketoacidosis or hyperosmolar hyperglycemic state who usually have a decreased LOS. The findings between glucose/lactate molar ratios show a novel representation of data that clearly shows that the patients in the first quartile (i.e., those with the highest lactate in proportion to glucose) have increased mortality. Interestingly, the LOS was not affected by this representation of the data.

Some of the strengths of this study include a large population sample, with patients included from multiple clinical settings and services. The authors recognize the limitations of a retrospective study, including the inability to discriminate stress-induced hyperglycemia from diabetes mellitus (lack of a glycosylated hemoglobin value), the lack of serial measurements of lactate and glucose values, and the inability to determine the causes of death.

## Conclusions

The combined use of glucose and lactate levels provides useful information for early-risk stratification to identify those patients with worse clinical outcomes. Patients with very high glucose and very high lactate levels need more evaluation and intensive care.
